# Steroid-Sparing Effect of Tocilizumab and Methotrexate in Patients with Polymyalgia Rheumatica: A Retrospective Cohort Study

**DOI:** 10.3390/jcm10132948

**Published:** 2021-06-30

**Authors:** Keisuke Izumi, Okinori Murata, Misako Higashida-Konishi, Yuko Kaneko, Hisaji Oshima, Tsutomu Takeuchi

**Affiliations:** 1 Division of Rheumatology, Department of Internal Medicine, Keio University School of Medicine, Tokyo 160-8582, Japan; okinori-nov.4@hotmail.co.jp (O.M.); ykaneko.z6@keio.jp (Y.K.); tsutake@z5.keio.jp (T.T.); 2 Department of Connective Tissue Diseases, National Hospital Organization Tokyo Medical Center, Tokyo 152-8902, Japan; higashidamisako@gmail.com (M.H.-K.); hoshimamac@mac.com (H.O.)

**Keywords:** glucocorticoid, methotrexate, polymyalgia rheumatica, retrospective study, interleukin-6

## Abstract

Polymyalgia rheumatica (PMR) is an inflammatory disorder characterized by pain and stiffness in the shoulders, hips, and proximal limbs; it usually affects elderly patients. The effectiveness of methotrexate and tocilizumab in PMR treatment has not been extensively studied. Thus, we aimed to assess the steroid-sparing effect of tocilizumab and methotrexate in PMR in clinical practice. Consecutive patients with PMR in our hospitals, who were included in our retrospective cohort, were reviewed between 2005 and 2015 and divided into the following groups according to their treatments: prednisolone or none (prednisolone group), methotrexate ± prednisolone (methotrexate group), or tocilizumab ± prednisolone (tocilizumab group). The prednisolone dose at the last follow-up was compared. A total of 227 patients with an average age of 74 years were enrolled. No difference in baseline characteristics was found among the three groups. The prednisolone dose at the last follow-up was lower (0 vs. 3.0 vs. 3.5 mg/day, *p* < 0.001) and the prednisolone discontinuation rate was higher (80.0% vs. 28.3% vs. 18.8%, *p* < 0.0001) in the tocilizumab group than in the prednisolone and methotrexate groups. This study suggested that tocilizumab has a steroid-sparing effect in PMR. Tocilizumab can be an option in the management of PMR. Future studies are warranted to confirm our findings.

## 1. Introduction

Polymyalgia rheumatica (PMR) is an inflammatory disorder characterized by severe pain and stiffness in the shoulders, hips, and proximal limbs; PMR usually affects elderly patients [1,2]. Despite acute and severe symptoms and laboratory systemic inflammation, low-to-moderate dose of systemic glucocorticoid therapy is quite effective [3,4].

Many patients (20–55%), however, will relapse during the disease course along with glucocorticoid tapering [1]. Long-term glucocorticoid use induces many adverse events, such as infection, diabetes mellitus, and osteoporosis, especially in elderly patients [5]; therefore, various immunosuppressive agents have been used to reduce the glucocorticoid dose. Methotrexate has been shown to be associated with shorter prednisone treatment duration and has steroid-sparing effects, but its effectiveness is controversial [6,7,8]. Tocilizumab, a monoclonal antibody to interleukin-6 receptor (IL-6R), is another treatment for PMR; recently, there have been promising studies on the cytokine profiles in PMR and the pilot use of tocilizumab in patients with PMR [9,10]. Tocilizumab reduces inflammation markers [11] and imaging findings [12] in PMR, and there are reports of its therapeutic [13] or steroid-sparing effects in PMR [14]. However, little is known about the effectiveness of methotrexate and tocilizumab in real-world setting, especially with a tapering steroid dose.

Therefore, this study aimed to assess the steroid-sparing effect of tocilizumab and methotrexate in PMR in clinical practice.

## 2. Materials and Methods

Patients with PMR who met the 2012 European League Against Rheumatism/American College of Rheumatology classification criteria [15] and had newly initiated glucocorticoid therapy between 2005 and 2015 at Keio University Hospital or National Tokyo Medical Center were retrospectively reviewed. Patients with (1) a final diagnosis of PMR at the time of their last visit, and (2) a treatment duration of more than six months in the hospital, were included. Patients with giant cell arteritis who met the 1990 American College of Rheumatology classification criteria [16], and those receiving concomitant methotrexate and tocilizumab, were excluded from the study. Our study was approved by the institutional review boards of the participating institutions (the approval numbers of Keio University Hospital and National Tokyo Medical Center were 20130404 and R14-007, respectively), and the requirement of patients’ written informed consent was waived according to the regulations in Japan.

The patients were divided into three groups according to the medication received at the last visit: prednisolone or none (prednisolone group), methotrexate ± prednisolone (methotrexate group), or tocilizumab ± prednisolone (tocilizumab group).

The primary outcome was the median prednisolone dose at the last follow-up. The secondary outcomes were the prednisolone discontinuation rate at the last visit, factors associated with prednisolone discontinuation, adverse events and comorbidities, and relapse. Continuous variables were compared using the Wilcoxon rank-sum test, and the proportion rates were compared using Fisher’s exact test. A two-sided *p*-value < 0.05 indicated statistical significance. JMP^®^ 14.3 (SAS Institute Inc., Cary, NC, USA) was used for all analyses. We used the last observation carried forward approach for imputing missing values in our study.

## 3. Results

### 3.1. Patients’ Characteristics

A total of 227 patients with PMR were identified. After excluding three patients who had been irresponsive to methotrexate and treated with tocilizumab add-on, 224 were included (prednisolone group (*N* = 177), methotrexate group (*N* = 32), and tocilizumab group (*N* = 15)). All the excluded three patients with concomitant methotrexate and tocilizumab could discontinue prednisolone. Two patients who had discontinued methotrexate because of nausea or pneumonia were included in the prednisolone group. Four patients who had switched from methotrexate to tocilizumab because of the ineffectiveness of methotrexate were included in the tocilizumab group. No patient discontinued tocilizumab. Rheumatoid factor was positive in 4.4% of patients with PMR. Anti-citrullinated peptide antibody was positive in 2.2% of patients with PMR.

Sex, age, body weight, and C-reactive protein (CRP) levels at PMR diagnosis were comparable among the three groups (Table 1). Prednisolone was initiated at a lower dose in the prednisolone group than in the other groups (15.0 (interquartile range, IQR: 10.0–15.0) vs. 15.0 (15.0–20.0) vs. 15.0 (15.0–20.0)). At the initiation of methotrexate or tocilizumab, the median age of patients was 76.4 years in both groups, the duration from diagnosis of PMR to the start of methotrexate and tocilizumab was 8.9 and 21.3 months, the CRP level was 0.75 and 0.86 mg/dL, and the prednisolone dose was 8.5 and 7.0 mg/day, respectively. The median dose of methotrexate was 8.0 mg/week, and tocilizumab was administered intravenously at a dose of 8 mg/kg every 4 weeks. The durations of methotrexate and tocilizumab use at the last visit were 26.6 and 21.0 months, respectively.

**Figure 1:**
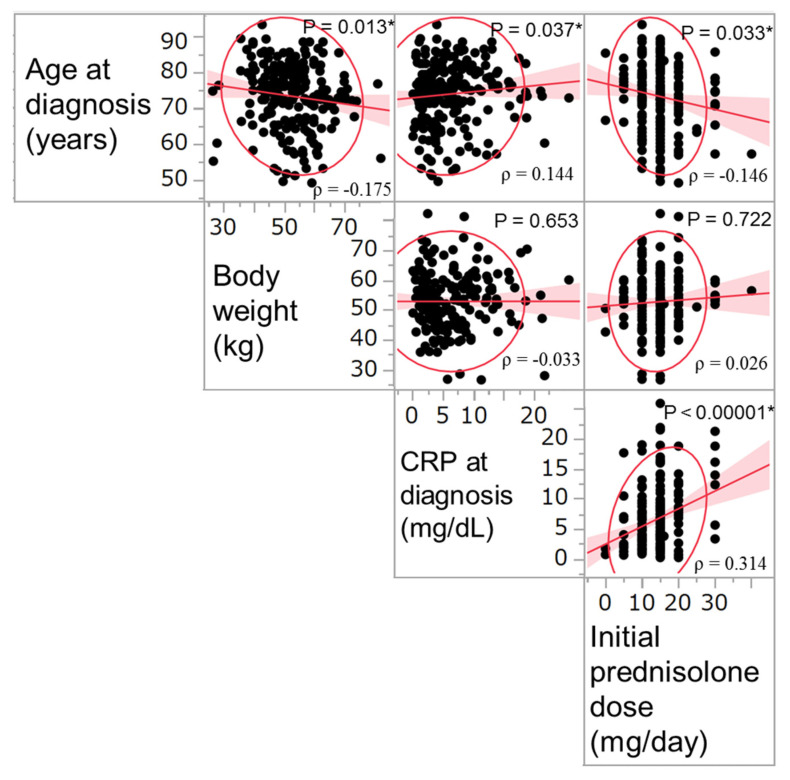
Correlations between the variables of age, body weight, CRP levels, and prednisolone dose at diagnosis (*N* = 224). Asterisks show statistical significance (*p* < 0.05). CRP, C-reactive protein.

The correlations between age, body weight, CRP levels, and prednisolone dose at diagnosis are shown in Figure 1. Older patients had lower body weight, higher levels of CRP at diagnosis, and lower initial dose of prednisolone. Patients with higher levels of CRP at diagnosis were administered a higher dose of initial prednisolone (*p* < 0.00001; rho = 0.314), regardless of their body weight.

### 3.2. Prednisolone Dose at the Last Visit

The prednisolone dose at the last visit was significantly higher in the prednisolone and methotrexate groups than in the tocilizumab group (3.0 vs. 3.5 vs. 0.0 mg/day, *p* < 0.0001, Table 1). A significantly lesser proportion of patients could discontinue prednisolone in the prednisolone and methotrexate groups than in the tocilizumab group (18.8% vs. 27.7% vs. 80.0%, *p* < 0.0001, Table 1).

### 3.3. Factors Associated with Prednisolone Discontinuation

Comparison of baseline characteristics and treatment regimens between patients who discontinued prednisolone and those who did not revealed that patients with prednisolone discontinuation had lower levels of CRP at diagnosis (*p* = 0.056) and had a lower initial dose of prednisolone (*p* = 0.048) (Table 2). Univariate and multivariate logistic regression analyses showed that initial prednisolone dose (*p* = 0.034) and tocilizumab use (*p* < 0.0001) were independent factors for discontinuation of prednisolone at the last visit (Table 3). When we focused on the prednisolone group, the analysis revealed that discontinuation of prednisolone at the last visit was associated with lower levels of CRP at diagnosis (*p* = 0.099) and lower initial dose of prednisolone (*p* = 0.040) (Table 4).

Then, we classified the patients by baseline CRP levels and initial prednisolone dose and evaluated the prednisolone discontinuation rates. In patients with CRP levels ≤5 mg/dL at the time of diagnosis, prednisolone discontinuation rates at the last visit were 83.3% in the tocilizumab group, 34.0% in the prednisolone group, and 33.3% in the methotrexate group. In patients with CRP levels >5 mg/dL at the time of diagnosis, while prednisolone discontinuation rates at the last visit were equally high in the tocilizumab group (77.9%), those in the prednisolone and methotrexate groups were very low (19.5% and 5.9%, respectively) (Table 5A). Similar results were observed when patients were categorized by initial prednisolone dose (Table 5B).

### 3.4. Adverse Events and Comorbidities

No serious adverse events were observed in the prednisolone and methotrexate groups. A serious adverse event associated with tocilizumab use was phlegmone (*N* = 1), but the patient did not discontinue tocilizumab. Comorbidities at the last visit in the three groups are shown in Table S1 (Supplementary Materials). Osteoporosis was observed in many patients, especially those in the tocilizumab group (prednisolone group, *N* = 102 (68.9%); methotrexate group, *N* = 21 (72.4%); tocilizumab group, *N* = 12 (85.7%)).

### 3.5. Relapses

During combination therapy, there were two relapses in the methotrexate group, whereas no relapse was found in the tocilizumab group at the last follow-up visit.

## 4. Discussion

Our study demonstrated that tocilizumab has a strong steroid-sparing effect compared to prednisolone alone or methotrexate in patients with PMR, with acceptable safety even in the elderly with an average age of 76 years in clinical practice.

We found that patients who discontinued prednisolone had lower levels of CRP at diagnosis than those who did not discontinue prednisolone (*p* = 0.056). Birra et al. reported that the CRP levels at 6 months of treatment were significantly lower in patients who achieved complete remission after 1 year than in patients who did not achieve complete remission [17]. They reported that in multivariate analysis, low CRP levels at 6 months were associated with complete remission at 12 months. In our study, we analyzed the baseline levels of CRP and thus, further analysis of any post-treatment changes in CRP levels must be conducted in the future study.

Given that IL-6 plays a critical role in PMR [10,18], IL-6 blocking has been a promising strategy for PMR. Methotrexate is known to reduce plasma IL-6 levels [19], and the European League Against Rheumatism/American College of Rheumatology recommended the concomitant use of methotrexate with prednisolone for PMR, particularly in patients at a high risk of relapse and/or prolonged therapy or in patients with relapse without significant response to glucocorticoids or experiencing glucocorticoid-related adverse events [20]; however, its effectiveness has been controversial [6,7,8]. A favorable effect of tocilizumab, a direct inhibitor of IL-6R, on PMR has been reported in several small studies [10,13,14,21], and two randomized trials on tocilizumab for PMR are being conducted (SEMAPHORE and PMR-SPARE trials). Our study revealed that patients using tocilizumab could be administered with a lower prednisolone dose than those treated with prednisolone alone or with additional methotrexate with few serious adverse events in real-world settings. Such data should be accumulated to optimize the management of PMR in the future.

A study by Tanaka et al. reported that lifetime costs and quality-adjusted life years (QALYs) were approximately 1.5 and 1.3 times higher, respectively, for tocilizumab treatment compared with methotrexate treatment in rheumatoid arthritis [22]. Thus, while tocilizumab is expensive, the increased QALYs may indicate cost-effectiveness, especially in patients with severe PMR or with side effects of prednisolone. Furthermore, the cost of tocilizumab is much lower than the other biologics in Japan.

Our results may be overstated because this study was retrospective in nature, a major limitation of the study, with a probable selection bias. To minimize selection bias, we performed propensity score matching for disease duration and initial dose of prednisolone (Table S2A–C), since correction for confounding variables such as disease duration and initial dose of prednisolone was performed. The results were the same with propensity score matching for disease duration and initial dose of prednisolone as without matching. Tocilizumab exhibited a strong steroid-sparing effect compared to prednisolone alone or methotrexate in patients with PMR. However, we were only able to include a limited number of cases, and further studies with a larger sample size are necessary.

The dose of methotrexate used in this study appeared low. However, because methotrexate concentrations are strongly affected by the body mass index (BMI) [23], therefore, this dose was reasonable because of the low body weight in our study (median, 53 kg). Furthermore, in our study, the average patient age was 76 years. Considering the low body weight and age, the average dose of 8 mg/week methotrexate might be the limit as the treatment of PMR in Japanese elderly patients from the viewpoint of safety.

Consequently, we believe that tocilizumab is a good option for refractory PMR with an acceptable concern of adverse effects.

## Figures and Tables

**Table 1:** Characteristics of the patients in the three groups.

	Prednisolone Group (*N* = 177)	Methotrexate Group (*N* = 32)	Tocilizumab Group (*N* = 15)	*p*-Values		
				PSL vs. MTX	PSL vs. TCZ	MTX vs. TCZ
**At diagnosis**						
Women, *N* (%)	130 (73.5)	25 (78.1)	11 (73.3)	0.57	0.99	0.53
Age, year	74.1 (67.5–79.5)	74.4 (64.0–79.7)	74.7 (66.0–83.5)	0.71	0.71	0.40
Body weight, kg	52.4 (47.0–59.2)	54.0 (44.3–59.2)	52.0 (42.5–60.7)	0.91	0.80	0.99
CRP, mg/dL	4.58 (2.30–8.31)	6.40 (3.60–10.46)	6.90 (3.35–10.33)	0.07	0.24	1.00
PSL dose, mg/day	15.0 (10.0–15.0)	15.0 (15.0–20.0)	15.0 (15.0–20.0)	0.02 *	0.06	0.90
**At the initiation of the combination therapy**						
Age, year	–	76.4 (68.1–81.9)	76.4 (67.4–84.8)	–	–	0.78
Disease duration, month	–	8.9 (3.6–41.7)	21.3 (3.6–35.8)	–	–	0.91
CRP, mg/dL	–	0.75 (0.20–1.71)	0.86 (0.10–1.24)	–	–	0.91
PSL dose, mg/day	–	8.5 (6.3–10.0)	7.0 (5.0–8.0)	–	–	0.08
**At the last follow-up**						
Disease duration, month	63.1 (25.0–111)	42.1 (20.5–88.8)	47.0 (24.0–59.4)	0.23	0.07	0.59
Combination therapy duration, month	–	26.6 (11.9–44.3)	21.0 (15.0–31.0)	–	–	0.59
Discontinuation of PSL, *N* (%)	49 (27.7)	6 (18.8)	12 (80.0)	0.38	<0.0001 *	<0.0001 *
PSL dose, mg/day	3.0 (0–5.0)	3.5 (1.6–6.0)	0 (0–0)	0.16	<0.001 *	<0.001 *

Data are presented as numbers (%) or median (IQR). An asterisk shows statistical significance (*p* < 0.05). CRP, C-reactive protein; IQR, interquartile range; PSL, prednisolone; MTX, methotrexate; TCZ, tocilizumab. Data of the initial prednisolone dose at the time of diagnosis were available in 208 patients.

**Table 2:** Patient characteristics stratified according to prednisolone discontinuation at the last visit.

	Prednisolone Discontinuation at the Last Visit		*p*-Value
	(+) *N* = 67	(−) *N* = 157	
Women, *N* (%)	47 (70.1)	119 (75.8)	0.407
Age at diagnosis, year	74.2 (66.3–80.4)	74.4 (66.9–79.6)	0.874
Body weight at diagnosis, kg	51.9 (45.1–60.0)	53.0 (47.0–59.0)	0.815
CRP at diagnosis, mg/dL	3.93 (2.23–7.35)	5.55 (3–9.14)	0.056
Initial prednisolone dose, mg/day	15.0 (10.0–15.0)	15.0 (10.0–15.0)	0.048 *
Prednisolone dose at the last visit, mg/day	0	4 (2.75–6)	<0.0001 *
Treatment group, *N* (%)	Prednisolone 49 (73.1) Methotrexate 6 (9.0) Tocilizumab 12 (17.9)	Prednisolone 128 (81.5) Methotrexate 26 (16.6) Tocilizumab 3 (1.9)	<0.0001 *
Observation period, months	56.1 (27.0–83.8)	59.2 (22.8–113.8)	0.479

Data are presented as numbers (%) or median (IQR). An asterisk shows statistical significance (*p* < 0.05). CRP, C-reactive protein; IQR, interquartile range. Mean (standard deviation) of the initial prednisolone dose in the prednisolone discontinuation (+) group and (−) group was 13.2 (3.85) and 14.9 (5.84) mg/day, respectively.

**Table 3:** Univariate and multivariate logistic regression analyses of factors for discontinuation of prednisolone at the last visit.

	Univariate Analysis		Multivariate Analysis	
	OR (95% CI)	*p*	OR (95% CI)	*p*
Sex (female/male)	0.7504 (0.3989–1.4367)	0.381		
Age at diagnosis, years	0.9948 (0.9634–1.0277)	0.753		
Body weight at diagnosis, kg	0.9961 (0.9633–1.0296)	0.816		
CRP at diagnosis, mg/dL	0.9433 (0.8784–1.0062)	0.077	0.9529 (0.9921–1.0293)	0.221
Initial prednisolone dose, mg/day	0.9348 (0.8772–0.9962)	0.028 *	0.9080 (0.8409–0.9805)	0.034 *
Disease duration at last visit, month	0.9982 (0.9920–1.0019)	0.248		
Tocilizumab use	11.200 (3.4081–50.500)	<0.0001 *	15.490 (3.1472–76.243)	<0.0001 *
Methotrexate use	0.4956 (0.1772–1.1938)	0.122		

Baseline factors with *p*-values < 0.1 in the univariate analysis were entered into the multivariate analysis. Asterisks (*) indicate *p* < 0.05 by the likelihood ratio test. CI, confidence interval; CRP, C-reactive protein; OR, odds ratio.

**Table 4:** Patient characteristics in the prednisolone group stratified according to prednisolone discontinuation at the last visit.

	Prednisolone Discontinuation at the Last Visit		*p*-Value
	(+) *N* = 49	(−) *N* = 128	
Women, *N* (%)	33 (67.4)	97 (75.8)	0.260
Age at diagnosis, year	73.0 (67.5–78.0)	74.4 (67.3–79.7)	0.529
Body weight at diagnosis, kg	51.6 (46.7–60.1)	52.7 (47.0–57.6)	0.941
CRP at diagnosis, mg/dL	3.76 (2.25–6.92)	5.15 (2.40–8.80)	0.099
Initial prednisolone dose, mg/day	10.0 (10.0–15.0)	15.0 (10.0–15.0)	0.040 *
Prednisolone dose at the last visit, mg/day	0	4 (2.5–6)	<0.0001 *
Disease duration at the last visit, months	61.0 (25.0–95.6)	63.1 (25.0–117.8)	0.521

Data are presented as numbers (%) or median (IQR). Asterisks show statistical significance (*p* < 0.05). CRP, C-reactive protein; IQR, interquartile range. Mean (standard deviation) of the initial prednisolone dose in the prednisolone discontinuation (+) group and (−) group was 12.6 (3.45) and 14.5 (5.71) mg/day, respectively.

**Table 5:** Prednisolone discontinuation rates at the last visit when patients were divided (**A**) by the CRP levels at diagnosis and (**B**) by initial prednisolone dose.

**(A) Prednisolone Discontinuation Rates in Patients with CRP Levels of >5 or ≤5 mg/dL at the Time of Diagnosis**						
**CRP Levels**	**Prednisolone Group**	**Methotrexate Group**	**Tocilizumab Group**	***p*-Values**		
				**Prednisolone vs. Methotrexate**	**Prednisolone vs. Tocilizumab**	**Methotrexate vs. Tocilizumab**
>5 mg/dL (*N* = 103), *N* (%)	15 (19.5)	1 (5.9)	7 (77.9)	0.288	0.001 *	<0.0001 *
≤5 mg/dL (*N* = 121), *N* (%)	34 (34.0)	5 (33.3)	5 (83.3)	1.000	0.025 *	0.063
**(B) Prednisolone Discontinuation Rates in the Patients with Initial Prednisolone Dose of ≥15 or <15 mg/day**						
**Initial Prednisolone Dose**	**Prednisolone Group**	**Methotrexate Group**	**Tocilizumab Group**	***p*-Values**		
				**Prednisolone vs. Methotrexate**	**Prednisolone vs. Tocilizumab**	**Methotrexate vs. Tocilizumab**
≥15 mg/day (*N* = 131), *N* (%)	21 (22.1)	4 (16.7)	9 (75.0)	0.551	0.0003 *	0.0005 *
<15 mg/day (*N* = 77), *N* (%)	25 (36.2)	1 (16.7)	2 (100)	0.307	0.046 *	0.022 *

Asterisks show statistical significance (*p* < 0.05). CRP, C-reactive protein.

## Data Availability

Not available.

## Supplementary Materials

The following are available online at https://www.mdpi.com/article/10.3390/jcm10132948/s1, Table S1: Comorbidities of the patients in the three groups at the last follow-up. Table S2A: Prednisolone dose and discontinuation of prednisolone at the last follow-up of the patients in the tocilizumab and prednisolone groups with propensity score matching for disease duration and initial dose of prednisolone. Table S2B: Prednisolone dose and discontinuation of prednisolone at the last follow-up of the patients in the tocilizumab and methotrexate groups with propensity score matching for disease duration and initial dose of prednisolone. Table S2C: Prednisolone dose and discontinuation of prednisolone at the last follow-up of the patients in the prednisolone and methotrexate groups with propensity score matching for disease duration and initial dose of prednisolone.
