# Supplementary material for: Steroid-Sparing Effect of Tocilizumab and Methotrexate in Patients with Polymyalgia Rheumatica: A Retrospective Cohort Study
Source: J Clin Med. 2021 Jun 30;10(13):2948. doi: 10.3390/jcm10132948 (PMC8267957; doi:10.3390/jcm10132948)
Supplement: Supplementary file 1 [file jcm-10-02948-s001.zip › jcm-1201334-supplementary.pdf]

Supplementary Material

Table S1. Comorbidities of the patients in the three groups at the last follow-up

	**Prednisolone group** (n=177)	**Methotrexate group** (n=32)	**Tocilizumab group** (n=15)
**Glucocorticoid-related comorbidities, N (%)**	136 (76.8)	24 (75.0)	13 (86.7)
**Osteoporosis*, N (%)**	102 (68.9)¶	21 (72.4)¶	12 (85.7) ¶
**Diabetes mellitus, N (%)**	45 (25.4)	9 (28.1)	2 (13.3)
**Hypertension, N (%)**	65 (36.7)	11 (34.4)	8 (53.3)
**Dyslipidemia, N (%)**	51 (28.8)	8 (25.0)	1 (6.67)

*Young adult mean bone mineral density < 70% (equivalent to T-score < -2.5)

¶Data of bone mineral density were available in 148 patients of the prednisolone group, 29 of the methotrexate group, and 14 of the tocilizumab

group.

Table S2A. Prednisolone dose and discontinuation of prednisolone at the last follow-up of the patients in the tocilizumab and prednisolone groups with propensity score matching for disease duration and initial dose of prednisolone

	TCZ (n=13)	PSL (n=13)	p
**PSL dose at the last follow-up, mg/day**	0	5 (0.3-6.8)	0.001*
**Discontinuation of PSL at the last follow-up, N (%)**	11 (84.6%)	3 (23.1%)	0.005*

PSL, prednisolone; TCZ, tocilizumab.

An asterisk shows statistical significance ($P < 0.05$).

Table S2B. Prednisolone dose and discontinuation of prednisolone at the last follow-up of the patients in the tocilizumab and methotrexate groups with propensity score matching for disease duration and initial dose of

prednisolone

	**TCZ (n=8)**	**MTX (n=8)**	**p**
**PSL dose at the last follow-up, mg/day**	0	3 (1.6-4.8)	0.006*
**Discontinuation of PSL at the last follow-up, N (%)**	7 (87.5%)	1 (12.5%)	0.01*

MTX, methotrexate; TCZ, tocilizumab.

An asterisk shows statistical significance ($P < 0.05$).

Table 2C. Prednisolone dose and discontinuation of prednisolone at the last follow-up of the patients in the prednisolone and methotrexate groups with propensity score matching for disease duration and initial dose of prednisolone

	**PSL (n=26)**	**MTX (n=26)**	**p**
**PSL dose at the last follow-up, mg/day**	3 (1.1-5.3)	3 (1.3-6.0)	0.87
**Discontinuation of PSL at the last follow-up, N (%)**	6 (23.1%)	5 (19.2%)	1.00

PSL, prednisolone; MTX, methotrexate.
